# Utility of Genetic Testing in Patients with Transthyretin Amyloid Cardiomyopathy: A Brief Review

**DOI:** 10.3390/biomedicines12010025

**Published:** 2023-12-21

**Authors:** Ana-Maria Merino-Merino, Jorge Labrador-Gomez, Ester Sanchez-Corral, Pedro-David Delgado-Lopez, Jose-Angel Perez-Rivera

**Affiliations:** 1 Cardiology Department, Universitary Hospital of Burgos, 09006 Burgos, Spain; esanchezco@saludcastillayleon.es (E.S.-C.); jangel.perezrivera@secardiologia.es (J.-A.P.-R.); 2 Haematology Department, Universitary Hospital of Burgos, 09006 Burgos, Spain; jlabradorg@saludcastillayleon.es; 3 Neurosurgery Department, Universitary Hospital of Burgos, 09006 Burgos, Spain; pdelgadol@saludcastillayleon.es; 4 Facultad de Ciencias de la Salud, Universidad Isabel I, 09003 Burgos, Spain

**Keywords:** transthyretin amyloidosis, genetics, family screening

## Abstract

Transthyretin amyloid cardiomyopathy (ATTR-CM) is an increasingly diagnosed condition. Although wild-type transthyretin amyloidosis (ATTRwt) is the most common ATTR-CM, hereditary transthyretin amyloidosis (ATTRv) may also occur. Currently, genetic testing for transthyretin pathogenic variants is recommended for patients with a confirmed clinical diagnosis of ATTR-CM. In fact, confirmation of this autosomal dominant pathogenic variant prompts genetic counselling and allows early identification of affected relatives. Additionally, in the presence of an ATTR-CM-associated polyneuropathy, specific drugs targeting transthyretin can be used. In this paper, we review the utility of genetic testing for the detection of pathogenic variants among patients harboring ATTR-CM and its impact on the natural history of the disease.

## 1. Introduction

Transthyretin amyloid cardiomyopathy (ATTR-CM) is an increasingly diagnosed condition causing heart failure. Although wild-type transthyretin amyloidosis (ATTRwt) is the most frequent form of ATTR-CM, hereditary transthyretin amyloidosis (ATTRv) can also occur [1]. ATTR is a rare disease resulting from the extracellular deposition of transthyretin (TTR) amyloid fibers. TTR is a transport protein produced especially in the liver but also in the retina and the choroid plexus. ATTRv is an autosomal dominant inherited disease that can occur as a result of more than 130 different mutations of the TTR gene [2]. The p.Val50Met mutation (previously p.Val30Met) was firstly identified, and it is the most common variant in Europe, whereas the p.Val142Ile mutation remains more prevalent among African Americans [3].

The exact prevalence of ATTRwt is unknown and likely underdiagnosed. It is estimated that around 13% of patients older than 60 years old have heart failure with preserved ejection fraction [4]. Recent evidence indicates that ATTR-CM is frequently disregarded as a source of prevalent cardiovascular diseases among elderly individuals, despite higher occurrence rates in cases of heart failure with preserved ejection fraction, low-flow aortic stenosis, and other circumstances involving augmented wall thickness [5]. Moreover, autopsy data have indicated that 25% of adults aged 80 years or older have significant TTR amyloid deposits in the myocardium [6]. Although ATTRwt has traditionally been viewed as a disease affecting older individuals, recent reports have indicated diagnosis in patients as young as 47 years of age [7].

The prevalence of ATTRv is much smaller (1/100,000 people) [8] and varies across countries and races. The penetrance of the disease is variable. Some of the variants are more prevalent in certain geographic areas due to the *founder effect*, like the p.Val50Met variant, especially prevalent in Portugal, Sweden, Japan, and the island of Majorca in Spain [9,10].

Currently, it is widely accepted that a non-invasive diagnosis of ATTR amyloidosis can be accomplished through imaging and nuclear tests. Specifically, a grade 2 or 3 cardiac uptake confirmed by diphosphonate scintigraphy, negative serum free light chains, and negative serum and urine immunofixation, when combined with echocardiographic or magnetic cardiac resonance criteria, serve as key factors in this diagnosis [1]. Signs and symptoms are known as “red flags” (Table 1).

**Figure 1:**
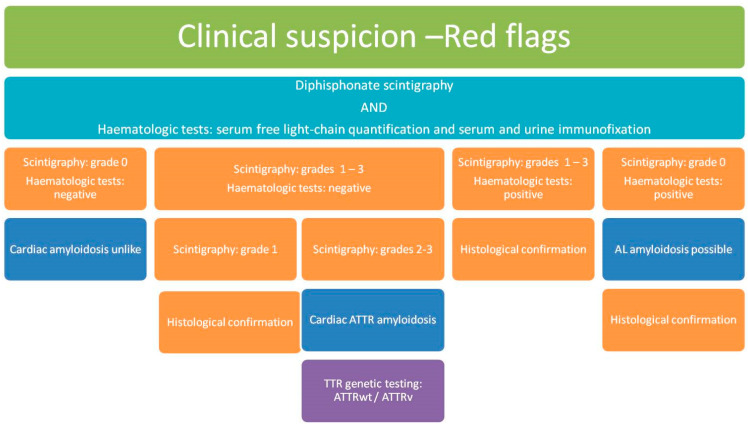
Diagnostic algorithm for cardiac amyloidosis. ATTR, transthyretin amyloidosis; ATTRv, hereditary transthyretin amyloidosis; ATTRwt, wild-type transthyretin amyloidosis; AL, light-chain amyloidosis; TTR, transthyretin. Adapted from: [4].

At the cardiac level, a diagnosis of left ventricular wall thickness greater than or equal to 12 mm and the presence of “red flags”, such as heart failure or aortic stenosis, particularly in individuals over 65 years old, are indicative of potential cardiac issues. Figure 1 shows the diagnostic algorithm for cardiac amyloidosis.

The age of onset in ATTRv varies from the second to the ninth decade of life [11]. Patients may present with neurologic symptoms (sensorimotor and autonomic neuropathy), cardiac involvement (infiltrative cardiomyopathy), and various nonspecific symptoms that may delay the diagnosis even for years [12]. Because of the low penetrance of the gene and the rare occurrence of de novo mutations, the diagnosis can be delayed by as many as four years, especially among patients without affected relatives [13].

At present, genetic testing aimed to differentiate between ATTRv and ATTRwt variants is recommended for all patients with a confirmed clinical diagnosis of ATTR-CM [1,14]. The diagnosis of a pathogenic variant prompts early identification of possible affected relatives (familial screening), allowing the use of specific TTR targeted therapies, especially in the presence of polyneuropathy [1]. In this paper, we review the overall utility of genetic testing in ATTR-CM patients.

As clinical or histologic techniques do not provide a means for reliable distinction between ATTRv and ATTRwt, TTR gene sequencing is advised for a conclusive diagnosis in all confirmed forms of ATTR-CM. Different societies recommend performing genetic testing, specifically for TTR pathogenic variants, for patients with restrictive cardiomyopathy and a clinical diagnosis of cardiac TTR amyloidosis (European Heart Rhythm Association (EHRA), Heart Rhythm Society (HRS), Asia Pacific Heart Rhythm Society (APHRS), Latin American Heart Rhythm Society (LAHRS)) [14]. The American Heart Association (AHA) states that if ATTR-CM is identified, then genetic sequencing of the TTR gene is required to define ATTRv versus ATTRwt [15].

Genetic testing for detecting TTR pathogenic variants is not established systematically in the vast majority of centres. In contrast, in an expert centre with an “Amyloid Team”, it is a usual test that is performed routinely. The need for a reference laboratory can be challenging and can be determinant for the centre to be able to have a test performed. However, it is not such an expensive test because there is only one gene that needs to be analysed. In Europe, there is an increasing development of reference laboratories that would be available for all the centres in a rapid way and with no extra cost in case the test is not available in the centre.

## 2. Utility of Family Screening

An early diagnosis of ATTRv is crucial for the patient and relatives. Early signs of the disease can be anticipated and monitored, and decisions regarding personal and family plans can be optimized and better informed [16]. All adult individuals at risk who may wish to undergo testing should be provided with updated and pertinent information to enable them to make a knowledgeable and self-determined decision. Pre-test counselling must encompass not just the complete process of testing but also the follow-up that occurs after the testing [17]. Genetic sequence analysis detects more than 99% of the causal mutations among patients carrying the ATTRv variant. However, no pathogenic variant can be detected by gene-targeted deletion/duplication analysis [18].

A study of Di Stefano et al. included 145 patients with sensory-motor idiopathic polyneuropathy and two or more “red flags” such as family history, bilateral carpal tunnel syndrome, and others. A genetic test was performed in all patients, and 14 of them (10%) had a positive test. Consecutively, a family screening discovered thirty-three carriers (seven symptomatic and twenty-six presymptomatic) [19].

According to Maestro-Benedicto et al. [20], in the elderly ATTR-CM population (older than 70), genetic testing identified the ATTRv variant in 35 out of 300 patients (12%) and nine additional cases among the patients’ relatives. These patients can secondarily benefit from active surveillance and eventual specific therapies at early stages of the disease. In the same line, another study [21] that included one hundred thirty patients older than 75 years identified eight patients (6.5%) carrying a pathogenic TTR variant. Specific treatment with tafamidis was initiated in four of these patients. Family screening was performed in 14 subjects. One of the subjects was diagnosed with ATTRv (with exclusive polyneuropathy involvement) and started on tafamidis. Six other asymptomatic carriers of the variant started the multidisciplinary follow-up. Both studies highlight the importance of performing a genetic test in an ATTR patient regardless of age.

An expert consensus statement has established the various clinical manifestations that can be considered the earliest detectable signs and symptoms in ATTRv amyloidosis [12]. The suggested protocol sets a standard for various crucial clinical measurements, determines the schedule and regularity of subsequent assessments based on an estimated age when the disease might commence, and pinpoints the initial clinical indicators and manifestations associated with the characteristics of the particular mutation in the TTR gene and the familial medical background. According to these authors, the specific type of mutation and its penetrance, as well as the age of onset of the affected relative, needs to be taken into account when determining the appropriate time to offer genetic testing to at-risk family members. In fact, both early- and late-onset ATTR amyloidosis can occur within the same family, and the phenotypic expression of a particular TTR genotypic subtype may vary even within affected members of the same family [12].

It has been proposed that clinical monitoring should begin at least 10 years before the predicted age of symptom onset. An initial reasonable follow-up scheme should include annual visits. Intervals between visits need to decrease as the expected date of onset approaches, especially for genotypes associated with a rapid progression (such as p.Ser43Asn). Efforts should also be made to identify early symptoms among relatives in order to initiate therapy to treat amyloidosis as soon as considered suitable.

The application of artificial intelligence to this field may improve the efficacy of the diagnosis of amyloidosis. García-García et al. [22] analysed the characteristics of this disease and proposed a statistical learning algorithm to aid in disease detection. Despite the limited number of amyloidosis patients in this study, the proposed approach demonstrated the ability to learn from clinical records even with limited data and showed proper validation in a sample of patients diagnosed with heart failure.

Another study [23] that included 184 patients with neuropathy and at least one “red flag” who were also eligible for genetic testing (probands) was conducted. The XGBoost (XGB) algorithm was trained to classify patients with positive and negative TTR mutations. The SHapley Additive exPlanations (SHAP) method was used as an explainable artificial intelligence algorithm to interpret the model’s findings. The XGB model demonstrated an accuracy of 0.707 ± 0.101, a sensitivity of 0.712 ± 0.147, a specificity of 0.704 ± 0.150, and an area under the receiving operating curve of 0.752 ± 0.107. According to the SHAP explanation, unexplained weight loss, gastrointestinal symptoms, and cardiomyopathy were significantly associated with the genetic diagnosis of ATTRv. In contrast, bilateral carpal tunnel syndrome, diabetes, autoimmunity, and ocular and renal involvement were associated with a negative genetic test.

In Europe, there is limited experience regarding prenatal and preimplantation diagnosis. In a Portuguese study, a sample of ATTRv mutation carriers aged 18 to 55 were questioned about their preferences regarding controlled reproduction, and this highlighted the importance of having accurate information on the matter and improved the accessibility to such reproductive techniques [24].

## 3. Utility of Genetic Testing in Clinical Characterization and Anticipation of Symptoms

Genetic counselling in the context of presymptomatic testing for late-onset diseases poses ethical and psychological issues that include the psychological stress of a patient who knows that they carry a pathogenic variant of the disease as well as the potential impact of this information on the reproductive and professional life of the patient and on his/her family relationships [16]. Once a specific mutation has been identified in a patient, a predicted age of disease onset can be estimated for their relatives [12].

For relatives considered to be at-risk, two pre-test counselling sessions are generally recommended. This is especially important for patients testing positive and approaching the expected date of onset and for those already presenting with mild symptoms [25]. In fact, the anticipation phenomenon is more common among patients inheriting mutations from the mother’s lineage [26].

In order to monitor asymptomatic TTR carriers, several protocols have been proposed [27]. The European Network for Transthyretin Familial Amyloid Polyneuropathy has identified five areas of assessment, including clinical examination, sensorimotor function, autonomic dysfunction, cardiac evaluation, and renal function [16].

**Figure 2:**
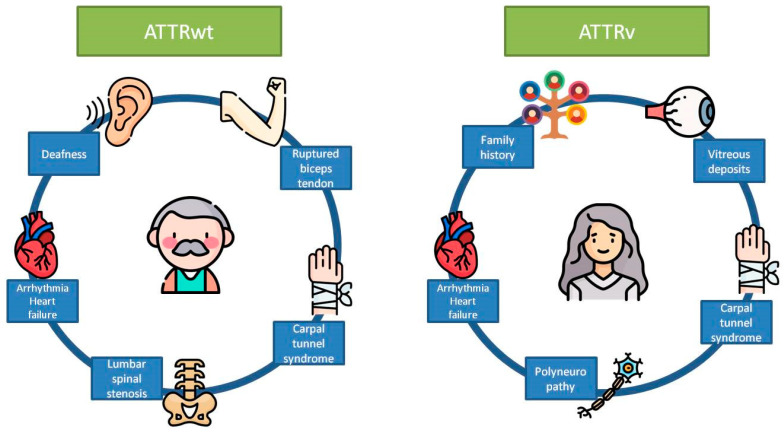
Major manifestations of the two subtypes of transthyretin-related cardiac amyloidosis. ATTRwt, wild-type transthyretin-related amyloidosis; ATTRv, variant transthyretin-related amyloidosis. Icons: Freepik fom https://www.flaticon.com/. Accessed on 1 November 2023.

Certain variants, like the p.Val50Met mutated subtype, usually produce neurological symptoms, whereas others, like p.Val142Ile, provoke late-onset cardiomyopathy with mild or no neurological involvement. However, most variants are associated with a mixed phenotype with both cardiac and neurological manifestations [28]. Globally, the ATTRv variant usually presents more as cardiomyopathy than polyneuropathy [16]. Despite that some variants have a predominant cardiac phenotype, they can also affect peripheral nerves, and expert neurologic assessment is recommended in all cases. Figure 2 shows the differences in symptoms between ATTRwt and ATTRv.

In Table 2, we show the most relevant features of the various TTR variants.

Conceição et al. [12] classify ATTR amyloidosis into four main phenotype groups: p.Val50Met early onset (neurological), p.Val50Met late onset (neurologic/mixed), non-p.Val50Met mixed phenotype, and non-p.Val50Met cardiac phenotype. They suggest performing a comprehensive evaluation to detect clinical changes from baseline at different intervals according to the group. Table 3 summarizes the recommended specific tests according to the presence or absence of the p.Val50Met variant.

In the French study by Damy et al. [40], out of 298 patients with increased left ventricular wall thickness, 15 (5%) showed ATTRv-CM. The authors compared the clinical, electrocardiogram, echocardiography, cardiac magnetic resonance, and biological baseline characteristics of the entire cohort regarding the TTR mutation and cardiac amyloidosis. They found that the mean left ventricular wall thickness was similar between the two groups. Additionally, African origin, male gender, carpal tunnel syndrome, neuropathy, a low voltage in the electrocardiogram, left ventricular enhancement using cardiac magnetic resonance, and a left ventricular ejection fraction less than 40% were significantly more frequent in ATTRv-CM versus non-ATTRv-CM. ATTRv-CM patients were also older on average. The authors highlighted the importance of molecular screening for this gene and suggested that the appearance of the hypertrophic cardiomyopathy phenotype in subjects with ATTRv is associated with late-stage disease. In fact, the management of ATTRv-CM patients differs from the management of those with hypertrophic cardiomyopathy caused by mutations in other genes.

The current protocol for the diagnosis of ATTR-CM needs to include grade 2 to 3 uptake using the 99mTc-DPD scintigraphy scan. However, in patients with an early-onset p.Val50Met mutation who present with neurologic symptoms, this test has been reported to show low sensitivity (as low as 41%) [41]. The diagnostic accuracy of bone scintigraphy for ATTR-CM has also been studied in other variants, such as p.Phe64Leu, where this approach showed low sensitivity (10.5%) and low accuracy (37%) [42]. The mechanisms by which bone tracers bind to the myocardium in TTR-CMare not yet fully understood. It has been hypothesised that the affinity of bone tracers to myocardial tissue may be related to the composition of amyloid fibrils [43].

## 4. Utility of Genetic Testing for the Initiation of a Specific Treatment

There is an increasing availability of novel, effective, and targeted therapeutic options for ATTRv, as well as for ATTRwt. As therapy is more effective in the early stages of the disease, a prompt diagnosis is key to enable the timely management of neurological, cardiac, and other systemic manifestations. The objective of such therapies is to decrease the production of overall and mutated TTR or to stabilize the circulating TTR molecule by preventing the dissociation of the molecule into amyloidogenic fragments [1].

Treatment alternatives vary according to the variant (ATTRwt versus ATTRv) and are also different within ATTRv patients, depending on the presence of cardiomyopathy, polyneuropathy, or both.

Tafamidis, a benzoxazole derivative without nonsteroidal anti-inflammatory activity, is a stabilizing molecule that selectively binds to the thyroxine-binding sites of TTR and inhibits the dissociation of tetramers into monomers. In the ATTR-ACT study, tafamidis was associated with a lower all-cause mortality than placebo and a lower rate of cardiovascular-related hospitalizations. It was also associated with a lower rate of decline in distance for the 6 minutes walk test and a lower rate of decline in the Kansas City Cardiomyopathy Questionnaire Overall Summary (KCCQ-OS) score [44]. Rapezzi et al. further analysed data from the ATTR-ACT in an attempt to determine whether the treatment effects were different between the ATTRv and ATTRwt groups. In fact, they found that the reduction in all-cause mortality following treatment with tafamidis compared with placebo was not different in the ATTRwt and ATTRv groups. In addition, tafamidis was associated with a similar reduction in the 6 min walk test distance and the KCCQ-OS score in both groups [45]. At present, tafamidis can be readily considered the agent of choice in both ATTRwt and ATTRv patients with a reasonable life expectancy who present with cardiac manifestations or stage 1 polyneuropathy [44]. Acoramidis is a new TTR stabilizer. Two phase 3 studies are ongoing to evaluate the efficacy and safety of acoramidis in patients with ATTR-CM (NCT03860935 and NCT04622046).

Ribonucleic acid (RNA) interference is an innate mechanism used to regulate gene expression. This process leads to the fragmentation of specific messenger RNA (mRNA) targets through the interaction of small interfering RNAs bound to the RNA-induced silencing complex. The genetic silencers decrease the production of transthyretin by targeting the transthyretin mRNA, causing the catalytic degradation of TTR mRNA in the liver. Patisiran and inotersen are two approved genetic silencers for ATTRv patients with polyneuropathy.

Patisiran is an interfering RNA encapsulated in a lipid nanoparticle that is delivered to the intracellular compartments of hepatocytes by intravenous infusion [46]. Patisiran has shown to improve multiple clinical manifestations of ATTRv. The favorable impacts of patisiran therapy on polyneuropathy and overall quality of life remained consistent across all primary subgroups, including age, gender, race, region, genotype, initial neuropathy impairment score, familial amyloid polyneuropathy stage, prior use of TTR stabilizers, and cardiac involvement, as observed in the APOLLO-A study [47]. Meanwhile, ongoing research in the APOLLO-B study (NCT03997383), targeting patients with ATTRwt and ATTRv associated with cardiomyopathy, aims to assess alterations in the 6 minuteswalk test from baseline at a 12-month interval. Patisiran presently serves as an alternative treatment for ATTRv patients with cardiac involvement, where gene silencers are used to manage neurological disease (specifically stage 1 or 2 polyneuropathy) [47]. Patisiran has also demonstrated benefits in orthostatic intolerance and gastrointestinal symptoms. In the APOLLO study, after 18 months of treatment, the patisiran group reported a 3.5-fold increase in improvement of diarrhea severity compared to the placebo group (18% versus 5%, respectively) [48].

Patisiran has also demonstrated that it is safe and effective in improving both the neurological and cardiovascular symptoms of ATTRv amyloidosis, and it can help patients maintain a good quality of life, regardless of the disease stage or the specific mutation involved [49].

Vutrisiran, a novel RNA interference agent, reduces serum TTR levels by curtailing TTR synthesis, and it has been recently approved for the treatment of ATTRv amyloidosis-related polyneuropathy in adults. This molecule is targeted to the liver, the primary site of TTR synthesis, by binding to a triantennary N-acetylgalactosamine ligand that attaches to the asialoglycoprotein receptor on hepatocyte surfaces. This binding design incorporates an enhanced stabilization chemistry, which enhances its potency and metabolic stability, thereby enabling subcutaneous injections once every three months [50]. The open-label, randomized study HELIOS-A compared the efficacy and safety of vutrisiran with an external placebo group (from the APOLLO study) in patients with ATTR amyloidosis presenting with polyneuropathy [51]. Patients were randomized to receive subcutaneous vutrisiran (25 mg) every three months or intravenous patisiran (0.3 mg/kg) every three weeks for eighteen months. Vutrisiran met the primary endpoint of modifying the Neuropathy Impairment Score +7 at nine months, along with all secondary efficacy endpoints. TTR reduction with vutrisiran was comparable to that observed with patisiran in the study. The majority of adverse events were mild or moderate in severity and aligned with the natural history of ATTRv amyloidosis. The impact of vutrisiran on the quality of life and physical function in patients with TTRv with polyneuropathy was also assessed [52]. This assessment revealed significant clinical benefits in various measures of quality of life and physical function in patients with ATTRv amyloidosis and polyneuropathy. These benefits were more pronounced in patients with earlier-stage disease, underscoring the importance of early detection and treatment. Additionally, a study investigating vutrisiran in cardiac amyloidosis (ATTRwt and ATTRv) is ongoing.

Antisense oligonucleotides (ASOs) are single-stranded amphipathic molecules that bind to proteins present in serum, on cell surfaces, and within cells. Within the nucleus of the target cell, ASOs bind to the target mRNA, initiating mRNA degradation [53].

Inotersen, a 2′-O-methoxyethyl–modified antisense oligonucleotide inhibitor, specifically targets the hepatic production of the transthyretin protein. Studies have indicated its ability to enhance the course of neurological disease and improve the quality of life in patients with hereditary transthyretin amyloidosis. These improvements were observed independently of the disease stage, mutation type, or the presence of cardiomyopathy [1,54]. Inotersen has also demonstrated a stabilization effect on the disease progression of ATTR cardiomyopathy patients [55]. Inotersen is a reasonable option for ATTRv patients with early-stage polyneuropathy involvement (stages 1 or 2 polyneuropathy) [46]. Gene silencers are only indicated for patients with ATTRv. The cost of these drugs, along with the cost of the genetic test, makes it difficult to generalise the use of these drugs for all healthcare systems.

Eplontersen represents a new generation of antisense oligonucleotide inhibitors that are linked to triantennary N-acetyl galactosamine. This linkage enhances uptake by hepatocytes, reducing immunogenic reactions and facilitating a reduction in TTR gene expression. Currently, a phase 3 study is underway to assess its effectiveness and safety in patients diagnosed with ATTR-CM (NCT04136171).

The application of genomic editing through CRISPR Cas9 is a promising therapeutic approach in managing ATTR amyloidosis. This technique involves using a nuclease (Cas9) combined with a single-stranded RNA, leading to the irreversible suppression of the TTR gene. A phase 1 clinical trial is ongoing in patients affected by ATTRv polyneuropathy, ATTRv, and ATTRwt CM (NCT04601051) [56].

Overall, patients diagnosed with stage 1 polyneuropathy survive longer [57]. Although therapy can delay disease progression, the recovery of established neurological deficits should not be expected [2]. At present, there is no consensus on what is considered the minimum level of organ damage needed to initiate treatment. This is important because classifying a carrier as affected usually prompts treatment initiation [2,16]. In general, disease-modifying therapies, like tafamidis, have significantly improved the survival of amyloidosis-affected patients [58].

## 5. Utility of Genetic Testing in Prognosis Assessment

The prognosis for ATTR amyloidosis exhibits variability based on factors such as mutation type, phenotype, and the delay in diagnosis [7]. Advances in diagnostic imaging, alongside the emergence of novel therapies targeting TTR, are contributing to an improved prognosis for ATTR-CM. In cases of ATTRwt and ATTRv with cardiac involvement, whether associated with neurological symptoms or not, the disease typically progresses, resulting in heart failure and mortality within approximately 10 years from the disease’s onset. However, in ATTRv patients with a pure neurological phenotype, the overall survival tends to be longer [59]. Specifically, the median survival time for untreated patients experiencing cardiomyopathy or a mixed cardiac-neurological presentation is estimated at 2.5 years for ATTRv and 3.6 years for ATTRwt [44].

Prognostic methods for cardiac amyloidosis patients include multiparametric biomarker scores and biomarker-based staging systems, primarily developed for AL and ATTR cardiac amyloidosis. Current scoring systems acknowledge several presentation parameters [1]. Gilmore et al. described a score for ATTRwt and ATTRv using glomerular filtration (< or >45 mL/min/1.73 m^2^) and NT-proBNP levels (< or >3000 pg/mL) [59]. Patients in stage 1 (zero parameters) had a median survival of 69.2 months, patients in stage 2 (one parameter) had a median survival of 46.7 months, and patients in stage 3 (two parameters) had a median survival of 24.1 months [1]. For the Mayo risk model [7], a group of ATTRwt patients underwent classification into three distinct prognostic stages determined by the levels of troponin T (>0.05 ng/mL) and N-terminal proB-type natriuretic peptide (>3000 pg/mL) biomarkers. Stage 1 comprised patients without elevated biomarkers, stage 2 included individuals with an elevation in one biomarker, and stage 3 encompassed those with elevations in both biomarkers. Patients in stage 3 exhibited a significantly poorer median survival compared to those in stage 1. The median survival times for patients in stages 1, 2, and 3 were 66, 40, and 20 months, respectively [60].

A study by Bézard et al. [61] included 648 patients with ATTR amyloidosis (423 ATTRwt and 225 ATTRv), 113 of which were treated with tafamidis (96 ATTRv and 17 ATTRwt). The mean age was 74 years. Patients with ATTRwt had a shorter median major cardiovascular outcome-free survival time than patients with ATTRv, regardless of their clinical presentation (cardiomyopathic, mixed cardiac-neurologic, or neurologic), for all three outcomes (cardiac decompensation, heart transplant, or death). For patients with ATTR-CM, treatment with tafamidis was associated with a longer median major cardiovascular outcome-free survival time compared with those not undergoing treatment (1565 (1010–2400) days in treated patients vs. 771 (686–895) days in non-treated patients (*p* < 0.001)).

A study by Ruberg et al. [62] included 29 patients: 18 with ATTRwt and 11 with ATTRv (p.Val142Ile was the predominant mutation). None of the patients were undergoing disease-modifying treatment, and the mean age was 74 years. They followed the patients for 2 years and found that the mortality rate of ATTRv patients was higher than that of ATTRwt patients (the median survival from diagnosis was 25.6 months for ATTRv vs. 43.0 months for ATTRwt (*p* = 0.04)), which was likely attributable to the marked prevalence of the p.Val142Ile variant in the sample.

A study by Álvarez-Rubio et al. [29] included 181 patients with ATTRv amyloidosis in which p.Val50Met was the most prevalent variant (67.8%), followed by p.Val142Ile (12.4%). The mean age at diagnosis was 62 years. The proportion of patients who underwent treatment with tafamidis was 25.4% and who underwent treatment with diflunisal was 24%, whereas 31.6% underwent a liver transplant and 4.5% underwent a heart transplant. After a median follow-up of 34.5 months, 60.2% required hospitalization mainly due to a cardiac (59.6%) or neurologic (17.4%) impairment: heart failure in the majority of patients (67%) belonging to the first group, and stroke among the majority of patients (57.8%) in the second group. Overall, one third (32.5%) of the patients died due to heart failure (28.8%), sudden cardiac death (11.5%), or a neurologic or renal cause (17.3%).

## 6. Conclusions

ATTR-CM is currently an increasingly diagnosed condition. ATTRwt is the most common variant, but hereditary subtypes may also occur. Genetic testing is generally recommended for all ATTR patients to detect such cases. The detection of an ATTRv mutated variant usually involves a multidisciplinary approach, including neurological assessments, and has implications regarding the initiation of currently available specific anti-amyloidotic therapies and family screening, both of which have the potential to impact the course of the disease.

## Figures and Tables

**Table 1:** Main cardiac and extracardiac red flags for ATTR amyloidosis.

Localization	Type	Red Flag	Amyloidosis Where It Is Most Frequently Found
Extracardiac	Clinical	Ruptured biceps tendon	ATTRwt
		Carpal tunnel syndrome	ATTRwt and ATTRv
		Deafness	ATTRwt
		Lumbar spinal stenosis	ATTRwt
		Polyneuropathy	ATTRv
		Vitreous deposits	ATTRv
		Family history	ATTRv
Cardiac	Clinical	Heart failure	ATTRwt and ATTRv
		Atrial fibrillation	ATTRwt and ATTRv
	ECG	Pseudoinfarct pattern	ATTRwt and ATTRv
		Low QRS voltage	ATTRwt and ATTRv
	Laboratory	Disproportionally elevated NT-proBNP and troponin	ATTRwt and ATTRv
	Echocardiogram	Granular sparkling of myocardium	ATTRwt and ATTRv
		Increased right ventricular wall thickness	ATTRwt and ATTRv
		Increased valve thickness	ATTRwt and ATTRv
		Pericardial effusion	ATTRwt and ATTRv
		Reduced longitudinal strain with apical sparing pattern	ATTRwt and ATTRv
	Cardiac magnetic resonance	Abnormal gadolinium kinetics	ATTRwt and ATTRv
		Elevated native T1 values	ATTRwt and ATTRv
		Increased extracellular volume	ATTRwt and ATTRv

ATTRwt, wild-type transthyretin-related amyloidosis; ATTRv, variant transthyretin-related amyloidosis. Modified from [1].

**Table 2:** Main variant characteristics.

Variant	Prevalence	Average Age of Onset in Years	Main Organs Involved	Prognosis	Reference
p.Val50Met	Unknown 1:10^6^ in Japan	<50	Neurologic (early onset) and neurologic/mixed (late onset)	Median of 28 years in early onset and 10 years in late onset	[29,30]
p.Val142Ile	0.3–1.6% in general population. Among African descent 3–3.5%.	7–8th decade of life	Cardiac	Median survival 3–5 years	[31]
p.Glu109Lys	50 patients described	40	Cardiac and neurologic predominant. Ophthalmologic in advanced.	Median life expectancy 61.6 years	[32]
p.Glu109Gln	Unknown	60	Neurologic and mixed	Unknown	[33]
p.Ser43Asn	Very rare. Unknown.	40–50	Cardiac	Before the age of 55	[34]
p.Thr60Ala	Unknown	63	Cardiac	Median survival 56.8 months	[35]
p.Val71Ala	Unknown	30–40	Cardiac and neurologic	Unknown	[36]
p.Tyr114Cys	Unknown	52	Neurologic	Unknown	[37]
p.Tyr134Ser	Unknown	65	Mixed	Survival time 10.6 years	[38]
p.Ala65Gly	Unknown	56–78	Late onset cardiomyopathy	Unknown	[39]

**Table 3:** Specific tests recommended according to the presence or absence of the p.Val50Met mutation.

	p.Val50Met	No p.Val50Met
Clinical evaluation	-NIS -BP supine/orthostatic -BMI	-BP supine/orthostatic -BMI
Neurophysiology	-NCS -Sudorimetry -HRDB -QST	-Not necessary
Biomarkers	-NT-proBNP -Troponin (only if late onset) -Blood sample	-NT-proBNP -Troponin -Blood sample
Cardiac evaluation	-Scintigraphy 99mTc-DPD (only if late onset) -Echo -ECG	-Scintigraphy 99mTc-DPD (only if late onset) -Echo -ECG -MRI

99mTc-DPD, technetium-99m-3,3-diphosphono-1,2 propanodicarboxylic acid; BMI, body mass index; BP, blood pressure; Echo, echocardiography; ECG, electrocardiography; HRDB, heart rate response to deep breathing; MRI, magnetic resonance imaging; NIS, Neuropathy Impairment Score; NCS, nerve conduction study; NT-proBNP, N-terminal pro-brain natriuretic peptide; QST, quantitative sensory testing.

## References

[1] Garcia-Pavia P., Rapezzi C., Adler Y., Arad M., Basso C., Brucato A., Burazor I., Caforio A.L.P., Damy T., Eriksson U. (2021). Diagnosis and Treatment of Cardiac Amyloidosis. A Position Statement of the European Society of Cardiology Working Group on Myocardial and Pericardial Diseases. Eur. J. Heart Fail..

[2] Ando Y., Coelho T., Berk J.L., Cruz M.W., Ericzon B.-G., Ikeda S., Lewis W.D., Obici L., Planté-Bordeneuve V., Rapezzi C. (2013). Guideline of Transthyretin-Related Hereditary Amyloidosis for Clinicians. Orphanet J. Rare Dis..

[3] Ueda M., Ando Y. (2014). Recent Advances in Transthyretin Amyloidosis Therapy. Transl. Neurodegener..

[4] González-López E., Gallego-Delgado M., Guzzo-Merello G., de Haro-Del Moral F.J., Cobo-Marcos M., Robles C., Bornstein B., Salas C., Lara-Pezzi E., Alonso-Pulpon L. (2015). Wild-Type Transthyretin Amyloidosis as a Cause of Heart Failure with Preserved Ejection Fraction. Eur. Heart J..

[5] Maurer M.S., Elliott P., Comenzo R., Semigran M., Rapezzi C. (2017). Addressing Common Questions Encountered in the Diagnosis and Management of Cardiac Amyloidosis. Circulation.

[6] Tanskanen M., Peuralinna T., Polvikoski T., Notkola I.-L., Sulkava R., Hardy J., Singleton A., Kiuru-Enari S., Paetau A., Tienari P.J. (2008). Senile Systemic Amyloidosis Affects 25% of the Very Aged and Associates with Genetic Variation in Alpha2-Macroglobulin and Tau: A Population-Based Autopsy Study. Ann. Med..

[7] Grogan M., Scott C.G., Kyle R.A., Zeldenrust S.R., Gertz M.A., Lin G., Klarich K.W., Miller W.L., Maleszewski J.J., Dispenzieri A. (2016). Natural History of Wild-Type Transthyretin Cardiac Amyloidosis and Risk Stratification Using a Novel Staging System. J. Am. Coll. Cardiol..

[8] González-López E., López-Sainz Á., Garcia-Pavia P. (2017). Diagnosis and Treatment of Transthyretin Cardiac Amyloidosis. Progress and Hope. Rev. Esp. Cardiol. (Engl. Ed.).

[9] Ohmori H., Ando Y., Makita Y., Onouchi Y., Nakajima T., Saraiva M.J.M., Terazaki H., Suhr O., Sobue G., Nakamura M. (2004). Common Origin of the Val30Met Mutation Responsible for the Amyloidogenic Transthyretin Type of Familial Amyloidotic Polyneuropathy. J. Med. Genet..

[10] Zaros C., Genin E., Hellman U., Saporta M.A., Languille L., Wadington-Cruz M., Suhr O., Misrahi M., Planté-Bordeneuve V. (2008). On the Origin of the Transthyretin Val30Met Familial Amyloid Polyneuropathy. Ann. Hum. Genet..

[11] Conceição I., González-Duarte A., Obici L., Schmidt H.H.-J., Simoneau D., Ong M.-L., Amass L. (2016). “Red-Flag” Symptom Clusters in Transthyretin Familial Amyloid Polyneuropathy. J. Peripher. Nerv. Syst..

[12] Conceição I., Damy T., Romero M., Galán L., Attarian S., Luigetti M., Sadeh M., Sarafov S., Tournev I., Ueda M. (2019). Early Diagnosis of ATTR Amyloidosis through Targeted Follow-up of Identified Carriers of TTR Gene Mutations. Amyloid.

[13] Planté-Bordeneuve V., Ferreira A., Lalu T., Zaros C., Lacroix C., Adams D., Said G. (2007). Diagnostic Pitfalls in Sporadic Transthyretin Familial Amyloid Polyneuropathy (TTR-FAP). Neurology.

[14] Wilde A.A.M., Semsarian C., Márquez M.F., Shamloo A.S., Ackerman M.J., Ashley E.A., Sternick E.B., Barajas-Martinez H., Behr E.R., Bezzina C.R. (2022). European Heart Rhythm Association (EHRA)/Heart Rhythm Society (HRS)/Asia Pacific Heart Rhythm Society (APHRS)/Latin American Heart Rhythm Society (LAHRS) Expert Consensus Statement on the State of Genetic Testing for Cardiac Diseases. Europace.

[15] Kittleson M.M., Maurer M.S., Ambardekar A.V., Bullock-Palmer R.P., Chang P.P., Eisen H.J., Nair A.P., Nativi-Nicolau J., Ruberg F.L., American Heart Association Heart Failure and Transplantation Committee of the Council on Clinical Cardiology (2020). Cardiac Amyloidosis: Evolving Diagnosis and Management: A Scientific Statement from the American Heart Association. Circulation.

[16] Obici L., Kuks J.B., Buades J., Adams D., Suhr O.B., Coelho T., Kyriakides T. (2016). European Network for TTR-FAP (ATTReuNET) Recommendations for Presymptomatic Genetic Testing and Management of Individuals at Risk for Hereditary Transthyretin Amyloidosis. Curr. Opin. Neurol..

[17] Manganelli F., Fabrizi G.M., Luigetti M., Mandich P., Mazzeo A., Pareyson D. (2022). Hereditary Transthyretin Amyloidosis Overview. Neurol. Sci..

[18] Sekijima Y., Adam M.P., Everman D.B., Mirzaa G.M., Pagon R.A., Wallace S.E., Bean L.J., Gripp K.W., Amemiya A. (1993). Hereditary Transthyretin Amyloidosis. GeneReviews^®^.

[19] Di Stefano V., Lupica A., Alonge P., Pignolo A., Augello S.M., Gentile F., Gagliardo A., Giglia F., Brinch D., Cappello M. (2024). Genetic Screening for Hereditary Transthyretin Amyloidosis with Polyneuropathy in Western Sicily: Two Years of Experience in a Neurological Clinic. Eur. J. Neurol..

[20] Maestro-Benedicto A., Vela P., de Frutos F., Mora N., Pomares A., Gonzalez-Vioque E., Briceño A., Cabrera E., Cobo-Marcos M., Dominguez F. (2022). Frequency of Hereditary Transthyretin Amyloidosis among Elderly Patients with Transthyretin Cardiomyopathy. Eur. J. Heart Fail..

[21] Gallego Delgado M., Gayán Ordás J., Eiros R., García Berrocal B., Sánchez P.L., Villacorta E. (2023). Importance of Genetic Study in Elderly Patients with Transthyretin Cardiac Amyloidosis. Med. Clin..

[22] García-García E., González-Romero G.M., Martín-Pérez E.M., de Zapata Cornejo E.D., Escobar-Aguilar G., Cárdenas Bonnet M.F. (2021). Real-World Data and Machine Learning to Predict Cardiac Amyloidosis. Int. J. Environ. Res. Public Health.

[23] Di Stefano V., Prinzi F., Luigetti M., Russo M., Tozza S., Alonge P., Romano A., Sciarrone M.A., Vitali F., Mazzeo A. (2023). Machine Learning for Early Diagnosis of ATTRv Amyloidosis in Non-Endemic Areas: A Multicenter Study from Italy. Brain Sci..

[24] Valdrez K., Silva S., Coelho T., Alves E. (2014). Awareness and Motives for Use and Non-Use of Preimplantation Genetic Diagnosis in Familial Amyloid Polyneuropathy Mutation Carriers. Prenat. Diagn..

[25] Sekijima Y. (2015). Transthyretin (ATTR) Amyloidosis: Clinical Spectrum, Molecular Pathogenesis and Disease-Modifying Treatments. J. Neurol. Neurosurg. Psychiatry.

[26] Lemos C., Coelho T., Alves-Ferreira M., Martins-da-Silva A., Sequeiros J., Mendonça D., Sousa A. (2014). Overcoming Artefact: Anticipation in 284 Portuguese Kindreds with Familial Amyloid Polyneuropathy (FAP) ATTRV30M. J. Neurol. Neurosurg. Psychiatry.

[27] Adams D., Cauquil C., Theaudin M., Rousseau A., Algalarrondo V., Slama M.S. (2014). Current and Future Treatment of Amyloid Neuropathies. Expert Rev. Neurother..

[28] Reinés J.B., Vera T.R., Martín M.U., Serra H.A., Campins M.M.C., Millán J.M.D., Lezaun C.G., Cruz M.R. (2014). Epidemiology of Transthyretin-Associated Familial Amyloid Polyneuropathy in the Majorcan Area: Son Llàtzer Hospital Descriptive Study. Orphanet J. Rare Dis..

[29] Álvarez Rubio J., Manovel Sánchez A.J., González-Costello J., García-Pavía P., Limeres Freire J., García-Pinilla J.M., Zorio Grima E., García-Álvarez A., Valverde Gómez M., Espinosa Castro M.Á. (2022). Characterization of Hereditary Transthyretin Cardiac Amyloidosis in Spain. Rev. Esp. Cardiol. (Engl. Ed.).

[30] González-Moreno J., Gaya-Barroso A., Losada-López I., Rodríguez A., Bosch-Rovira T., Ripoll-Vera T., Usón M., Figuerola A., Descals C., Montalà C. (2021). Val50Met Hereditary Transthyretin Amyloidosis: Not Just a Medical Problem, but a Psychosocial Burden. Orphanet J. Rare Dis..

[31] Chandrashekar P., Alhuneafat L., Mannello M., Al-Rashdan L., Kim M.M., Dungu J., Alexander K., Masri A. (2021). Prevalence and Outcomes of p.Val142Ile TTR Amyloidosis Cardiomyopathy: A Systematic Review. Circ. Genom. Precis. Med..

[32] de Frutos F., Ochoa J.P., Gómez-González C., Reyes-Leiva D., Aróstegui J.I., Casasnovas C., Barriales-Villa R., Sevilla T., Gonzalez-Lopez E., Ramil E. (2022). Phenotype and Clinical Outcomes of Glu89Lys Hereditary Transthyretin Amyloidosis: A New Endemic Variant in Spain. Amyloid.

[33] González-Moreno J., Losada-López I., Cisneros-Barroso E., Garcia-Pavia P., González-Costello J., Muñoz-Beamud F., Campistol J.M., Fernandez-Torron R., Chapman D., Amass L. (2021). A Descriptive Analysis of ATTR Amyloidosis in Spain from the Transthyretin Amyloidosis Outcomes Survey. Neurol. Ther..

[34] Papathanasiou M., Carpinteiro A., Kersting D., Jakstaite A.-M., Hagenacker T., Schlosser T.-W., Rischpler C., Rassaf T., Luedike P. (2021). Rare Variant (p.Ser43Asn) of Familial Transthyretin Amyloidosis Associated with Isolated Cardiac Phenotype: A Case Series with Literature Review. Mol. Genet. Genom. Med..

[35] Swiecicki P.L., Zhen D.B., Mauermann M.L., Kyle R.A., Zeldenrust S.R., Grogan M., Dispenzieri A., Gertz M.A. (2015). Hereditary ATTR Amyloidosis: A Single-Institution Experience with 266 Patients. Amyloid.

[36] Puffer R.C., Spinner R.J., Bi H., Sharma R., Wang Y., Theis J.D., McPhail E.D., Poterucha J.J., Niu Z., Klein C.J. (2019). Fatal TTR Amyloidosis with Neuropathy from Domino Liver p.Val71Ala Transplant. Neurol. Genet..

[37] Chaves M., Bettini M., Marciano S., Sáez S., Cristiano E., Rugiero M. (2016). Presentations of transthyretin associated familial amyloid polyneuropathy in Argentina. Medicina.

[38] Nakase T., Yamashita T., Matsuo Y., Nomura T., Sasada K., Masuda T., Misumi Y., Takamatsu K., Oda S., Furukawa Y. (2019). Hereditary ATTR Amyloidosis with Cardiomyopathy Caused by the Novel Variant Transthyretin Y114S (p.Y134S). Intern. Med..

[39] Klaassen S.H.C., Lemmink H.H., Bijzet J., Glaudemans A.W.J.M., Bos R., Plattel W., van den Berg M.P., Slart R.H.J.A., Nienhuis H.L.A., van Veldhuisen D.J. (2017). Late Onset Cardiomyopathy as Presenting Sign of ATTR A45G Amyloidosis Caused by a Novel TTR Mutation (p.A65G). Cardiovasc. Pathol..

[40] Damy T., Costes B., Hagège A.A., Donal E., Eicher J.-C., Slama M., Guellich A., Rappeneau S., Gueffet J.-P., Logeart D. (2016). Prevalence and Clinical Phenotype of Hereditary Transthyretin Amyloid Cardiomyopathy in Patients with Increased Left Ventricular Wall Thickness. Eur. Heart J..

[41] Rodrigues P., Dias Frias A., Gouveia P., Trêpa M., Fontes Oliveira M., Costa R., Reis H., Amorim I., Palma P., Cyrne Carvalho H. (2021). Radionuclide Imaging in the Diagnosis of Transthyretin Cardiac Amyloidosis: Different Sensitivity in Early-Onset V30M Mutation?. JACC Cardiovasc. Imaging.

[42] Musumeci M.B., Cappelli F., Russo D., Tini G., Canepa M., Milandri A., Bonfiglioli R., Di Bella G., My F., Luigetti M. (2020). Low Sensitivity of Bone Scintigraphy in Detecting Phe64Leu Mutation-Related Transthyretin Cardiac Amyloidosis. JACC Cardiovasc. Imaging.

[43] Rapezzi C., Gagliardi C., Milandri A. (2019). Analogies and Disparities among Scintigraphic Bone Tracers in the Diagnosis of Cardiac and Non-Cardiac ATTR Amyloidosis. J. Nucl. Cardiol..

[44] Maurer M.S., Schwartz J.H., Gundapaneni B., Elliott P.M., Merlini G., Waddington-Cruz M., Kristen A.V., Grogan M., Witteles R., Damy T. (2018). Tafamidis Treatment for Patients with Transthyretin Amyloid Cardiomyopathy. N. Engl. J. Med..

[45] Rapezzi C., Elliott P., Damy T., Nativi-Nicolau J., Berk J.L., Velazquez E.J., Boman K., Gundapaneni B., Patterson T.A., Schwartz J.H. (2021). Efficacy of Tafamidis in Patients with Hereditary and Wild-Type Transthyretin Amyloid Cardiomyopathy: Further Analyses From ATTR-ACT. JACC Heart Fail..

[46] Mathew V., Wang A.K. (2019). Inotersen: New Promise for the Treatment of Hereditary Transthyretin Amyloidosis. Drug Des. Dev. Ther..

[47] Adams D., Gonzalez-Duarte A., O’Riordan W.D., Yang C.-C., Ueda M., Kristen A.V., Tournev I., Schmidt H.H., Coelho T., Berk J.L. (2018). Patisiran, an RNAi Therapeutic, for Hereditary Transthyretin Amyloidosis. N. Engl. J. Med..

[48] González-Duarte A., Berk J.L., Quan D., Mauermann M.L., Schmidt H.H., Polydefkis M., Waddington-Cruz M., Ueda M., Conceição I.M., Kristen A.V. (2020). Analysis of Autonomic Outcomes in APOLLO, a Phase III Trial of the RNAi Therapeutic Patisiran in Patients with Hereditary Transthyretin-Mediated Amyloidosis. J. Neurol..

[49] Di Stefano V., Fava A., Gentile L., Guaraldi P., Leonardi L., Poli L., Tagliapietra M., Vastola M., Fanara S., Ferrero B. (2022). Italian Real-Life Experience of Patients with Hereditary Transthyretin Amyloidosis Treated with Patisiran. Pharmacogenomics Pers. Med..

[50] Habtemariam B.A., Karsten V., Attarwala H., Goel V., Melch M., Clausen V.A., Garg P., Vaishnaw A.K., Sweetser M.T., Robbie G.J. (2021). Single-Dose Pharmacokinetics and Pharmacodynamics of Transthyretin Targeting N-Acetylgalactosamine-Small Interfering Ribonucleic Acid Conjugate, Vutrisiran, in Healthy Subjects. Clin. Pharmacol. Ther..

[51] Adams D., Tournev I.L., Taylor M.S., Coelho T., Planté-Bordeneuve V., Berk J.L., González-Duarte A., Gillmore J.D., Low S.-C., Sekijima Y. (2023). Efficacy and Safety of Vutrisiran for Patients with Hereditary Transthyretin-Mediated Amyloidosis with Polyneuropathy: A Randomized Clinical Trial. Amyloid.

[52] Obici L., Ajroud-Driss S., Lin K.-P., Berk J.L., Gillmore J.D., Kale P., Koike H., Danese D., Aldinc E., Chen C. (2023). Impact of Vutrisiran on Quality of Life and Physical Function in Patients with Hereditary Transthyretin-Mediated Amyloidosis with Polyneuropathy. Neurol. Ther..

[53] Griffin J.M., Rosenthal J.L., Grodin J.L., Maurer M.S., Grogan M., Cheng R.K. (2021). ATTR Amyloidosis: Current and Emerging Management Strategies: JACC: CardioOncology State-of-the-Art Review. JACC CardioOncology.

[54] Benson M.D., Waddington-Cruz M., Berk J.L., Polydefkis M., Dyck P.J., Wang A.K., Planté-Bordeneuve V., Barroso F.A., Merlini G., Obici L. (2018). Inotersen Treatment for Patients with Hereditary Transthyretin Amyloidosis. N. Engl. J. Med..

[55] Benson M.D., Dasgupta N.R., Rissing S.M., Smith J., Feigenbaum H. (2017). Safety and Efficacy of a TTR Specific Antisense Oligonucleotide in Patients with Transthyretin Amyloid Cardiomyopathy. Amyloid.

[56] Monda E., Bakalakos A., Rubino M., Verrillo F., Diana G., De Michele G., Altobelli I., Lioncino M., Perna A., Falco L. (2023). Targeted Therapies in Pediatric and Adult Patients With Hypertrophic Heart Disease: From Molecular Pathophysiology to Personalized Medicine. Circ. Heart Fail..

[57] Russo M., Gentile L., Di Stefano V., Di Bella G., Minutoli F., Toscano A., Brighina F., Vita G., Mazzeo A. (2021). Use of Drugs for ATTRv Amyloidosis in the Real World: How Therapy Is Changing Survival in a Non-Endemic Area. Brain Sci..

[58] Falcão de Campos C., Conceição I. (2023). Updated Evaluation of the Safety, Efficacy and Tolerability of Tafamidis in the Treatment of Hereditary Transthyretin Amyloid Polyneuropathy. Drug Healthc. Patient Saf..

[59] Gillmore J.D., Damy T., Fontana M., Hutchinson M., Lachmann H.J., Martinez-Naharro A., Quarta C.C., Rezk T., Whelan C.J., Gonzalez-Lopez E. (2018). A New Staging System for Cardiac Transthyretin Amyloidosis. Eur. Heart J..

[60] Obi C.A., Mostertz W.C., Griffin J.M., Judge D.P. (2022). ATTR Epidemiology, Genetics, and Prognostic Factors. Methodist Debakey Cardiovasc. J..

[61] Bézard M., Kharoubi M., Galat A., Poullot E., Guendouz S., Fanen P., Funalot B., Moktefi A., Lefaucheur J.-P., Abulizi M. (2021). Natural History and Impact of Treatment with Tafamidis on Major Cardiovascular Outcome-Free Survival Time in a Cohort of Patients with Transthyretin Amyloidosis. Eur. J. Heart Fail..

[62] Ruberg F.L., Maurer M.S., Judge D.P., Zeldenrust S., Skinner M., Kim A.Y., Falk R.H., Cheung K.N., Patel A.R., Pano A. (2012). Prospective Evaluation of the Morbidity and Mortality of Wild-Type and V122I Mutant Transthyretin Amyloid Cardiomyopathy: The Transthyretin Amyloidosis Cardiac Study (TRACS). Am. Heart J..

